# Characterization of the Noncanonical Regulatory and Transporter Genes in Atratumycin Biosynthesis and Production in a Heterologous Host

**DOI:** 10.3390/md17100560

**Published:** 2019-09-29

**Authors:** Zhijie Yang, Xin Wei, Jianqiao He, Changli Sun, Jianhua Ju, Junying Ma

**Affiliations:** 1 CAS Key Laboratory of Tropical Marine Bio-resources and Ecology, Guangdong Key Laboratory of Marine Materia Medica, RNAM Center for Marine Microbiology, South China Sea Institute of Oceanology, Chinese Academy of Sciences, Guangzhou 510301, China; yzjie6@126.com (Z.Y.); lingluboxi@163.com (C.S.); 2 College of Earth and Planetary Sciences, University of Chinese Academy of Sciences, Beijing 100049, China

**Keywords:** atratumycin, LuxR, SARP, ABC transporter, heterologous expression

## Abstract

Atratumycin is a cyclodepsipeptide with activity against *Mycobacteria tuberculosis* isolated from deep-sea derived *Streptomyces atratus* SCSIO ZH16NS-80S. Analysis of the atratumycin biosynthetic gene cluster (*atr*) revealed that its biosynthesis is regulated by multiple factors, including two LuxR regulatory genes (*atr1* and *atr2*), two ABC transporter genes (*atr29* and *atr30*) and one *Streptomyces* antibiotic regulatory gene (*atr32*). In this work, three regulatory and two transporter genes were unambiguously determined to provide positive, negative and self-protective roles during biosynthesis of atratumycin through bioinformatic analyses, gene inactivations and *trans*-complementation studies. Notably, an unusual *Streptomyces* antibiotic regulatory protein Atr32 was characterized as a negative regulator; the function of Atr32 is distinct from previous studies. Five over-expression mutant strains were constructed by rational application of the regulatory and transporter genes; the resulting strains produced significantly improved titers of atratumycin that were ca. 1.7–2.3 fold greater than wild-type (WT) producer. Furthermore, the atratumycin gene cluster was successfully expressed in *Streptomyces coelicolor* M1154, thus paving the way for the transfer and recombination of large DNA fragments. Overall, this finding sets the stage for understanding the unique biosynthesis of pharmaceutically important atratumycin and lays the foundation for generating anti-tuberculosis lead compounds possessing novel structures.

## 1. Introduction

Marine-derived natural products represent a rich source of potential drug candidates. Over the past 50 years, more than 30,000 natural products have been discovered from the ocean and unique marine microbes have become a treasure trove of novel lead compounds [1,2]. At the same time, tuberculosis (TB), a chronic and lethal disease caused by *Mycobacterium tuberculosis* has been a major threat worldwide and continues to impose heavy tolls upon humanity. Recently, increases in TB co-infection rates with HIV and the proliferation of multi-drug resistant bacteria have severely limited the effectiveness of conventional antibiotics, making it urgent to identify new anti-TB drug candidates [3]. In our efforts to find anti-TB drugs from marine-derived *Streptomyces*, the microbe *Streptomyces atratus* SCSIO ZH16 was found to generate a number of natural products with good activity against *M. tuberculosis*. For example, ilamycin and its derivatives produced by the *S. atratus* SCSIO ZH16 have notably specific anti-TB activities [4]. Interestingly, the novel compound atratumycin, identified originally from genome mining of *S. atratus* SCSIO ZH16NS-80S, a genetically engineered variant of *S. atratus* SCSIO ZH16, also has been found to possess significant anti-TB activities [5].

Our previous study showed that atratumycin is a cyclodecapeptide containing a unique cinnamic acid unit. The 2-dimensional structure and stereochemistry of atratumycin were determined using extensive spectroscopic data including those generated by X-ray crystallography and Marfey’s method analyses. Atratumycin was found to have minimal inhibitory concentration (MIC) values of 3.8 and 14.6 μM in assays against *M. tuberculosis* H37Ra and H37Rv, respectively [5]. Moreover, the biosynthetic gene cluster (BGC) of atratumycin (*atr*) was identified and the cluster boundaries were determined. Additionally, the only cytochrome p450 in the *atr* cluster has been characterized using both in vivo and in vitro methods in our laboratories. Careful analysis of the *atr* cluster revealed 33 genes in all; three appear to play assorted regulatory roles whereas two appear to be responsible for atratumycin transport [5]. Notably, the details of these regulatory and transporter genes have been, thus far, elusive.

We report here the functional characterization of the *atr* regulatory and transporter genes by way of in silico analyses as well as gene inactivations, *trans-*complementations and over-expression experiments. Two LuxR family regulators and two ABC transporter proteins have been identified and characterized herein. Over-expression of four LuxR or transporter genes significantly increased atratumycin production by 1.7–2.0 fold relative to wild-type (WT) strain, suggesting that they play positive roles in atratumycin production. Interestingly, the disruption of *atr32*, a peculiar *Streptomyces* antibiotic regulatory protein (SARP) gene, increased the yield of atratumycin by 2.3-fold, which was distinct from previous studies. In addition, a large vector containing the complete *atr* cluster was constructed and successfully expressed in the heterologous host *Streptomyces coelicolor* M1154. These achievements represent important steps in realizing improved atratumycin availability and access to related congeners via combinatorial biosynthetic strategies.

## 2. Results

### 2.1. In Silico Analysis of Regulatory Genes in the Atr BGC

**Figure 1:**
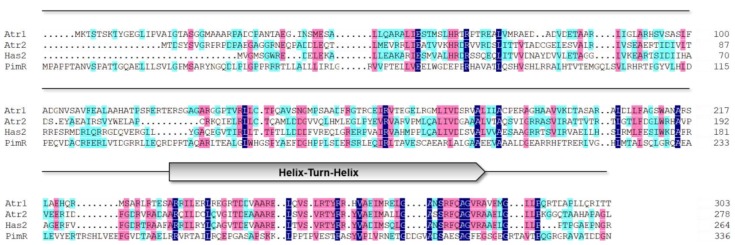
Multiple sequence alignment of Atr1 and Atr2 with Has2 (ALF39547.1, *Streptomyces* sp. LZ35) and PimR (CAE51066.1, *Streptomyces natalensis*).

Secondary metabolite production is one of the basic processes in *Streptomyces* and is carefully controlled by subtle and precise regulatory systems [6]. Regulatory and transporter proteins are integral to controlling the biosynthesis of antibiotics. Two LuxR family regulators (Atr1 and Atr2), two ABC transporters (Atr29 and Atr30) and one SARP regulator (Atr32) were found to reside in both upstream and downstream regions of the *atr* gene cluster. To characterize the function of the LuxR family regulators in production, a widely used in silico analysis was performed. LuxR family transcriptional regulators are key players in important physiological functions and are widely involved in biological processes such as quorum sensing and modulating secondary metabolism [7]. There are two typical functional domains in LuxR family regulators, a signal-binding domain in the N-terminal and a DNA-binding domain with a classical Helix-Turn-Helix (HTH) domain in the C-terminal region [8]. In general, LuxR family proteins bind to DNA in a dimeric form and specifically recognize hallmark sequences of given target gene’s regulatory region [9]. By aligning the amino acid sequences of Atr1 and Atr2 with the LuxR family, including Has2 (ALF39547.1, *Streptomyces* sp. LZ35) and PimR (CAE51066.1, *Streptomyces natalensis*), the results revealed that the HTH motifs of the LuxR family were highly conserved (Figure 1), which is mainly responsible for the protein multi-polarization and activation with gene promoters [10,11]. Even though the primary structure of LuxR was quite different, the advanced structure of LuxR was roughly the same enough to perform its positive regulation [12].

The SARP family proteins are generally universal pathway-specific regulators in *Streptomyces*. Atr32 was annotated as a SARP family transcriptional regulator protein; the full-length protein contains 667 amino acids. The secondary structure prediction indicated that Atr32, like other SARP family proteins, harbors two characteristic domains: the OmpR family at the N-terminal and the transcriptional activation domain (BTAD) located at the C-terminal region [13,14]. In addition, Atr32 contains a functional ATPase domain nucleotide-binding adaptor shared by APAF-1, R proteins and CED-4 (NB-ARC), which is generally considered to act as a molecular switch that toggles between active ATP-bound and inert ADP-bound forms [15].

**Figure 2:**
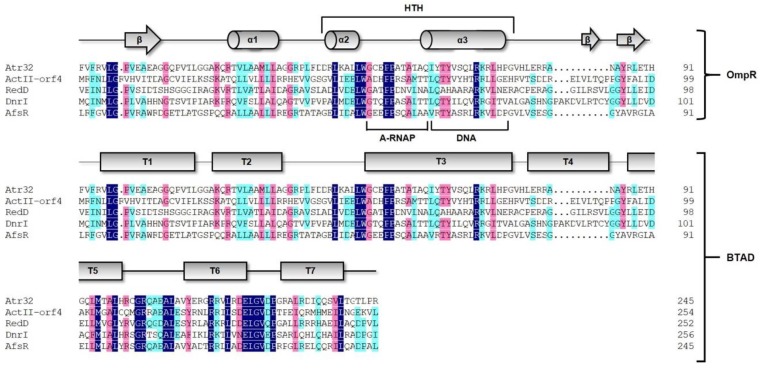
Multiple sequence alignment of Atr32 and ActII-ORF4, RedD, DnrI and AfsR.

To compare the amino acid sequence characteristics, other SARP regulators such as ActII-ORF4 (AAK32147.1, *Streptomyces coelicolor* A3(2)), a regulator of actinic purine production, DnrI (AAA26736.1, *Streptomyces peucetius*), a regulator of daunorubicin biosynthesis, RedD (AAA88556.1, *Streptomyces coelicolor* A3(2)), a regulator of undecyl erythromycin assembly, and the multi-effect regulatory factor AfsR (BAA14186.1, *Streptomyces coelicolor* A3(2)) were aligned and putative active motifs of the proteins were predicted by analysis of the conserved domains (Figure 2). These analyses revealed that the OmpR-like domain likely contains three alpha helices (α1, α2 and α3) whereas the α2, α3 and 10 residue loops form an HTH domain and the W1 and W2 flanks are composed of β-sheets at both ends [16,17]. The α2 helix acts as a scaffold and the α3 helix may be responsible for recognizing the major groove of DNA [16]. The amino acids between the α2 and α3 helices may interact with the alpha subunit of RNA polymerase (RNAP). In addition to other homologous proteins, the BTAD of Atr32 is also composed of seven tetratrico peptide repeats containing intact conserved cores (T1–T7), this architecture is required for functional SARP family proteins [18].

### 2.2. In Silico Analysis of Transporter Genes in the Atr BGC

**Figure 3:**
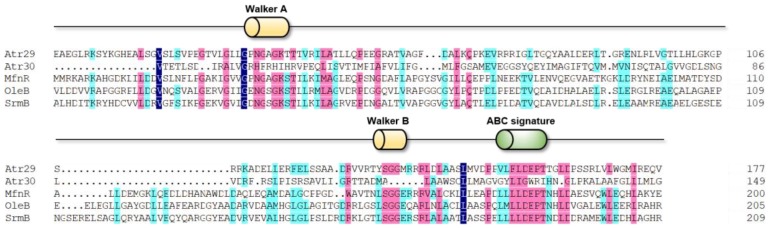
Multiple sequence alignment of Atr32 and other SARP family proteins. Mfn (AJV88390.1, *Streptomyces drozdowiczii*); OleB (AAA50325.1, *Streptomyces antibioticus*) and Srm (WP_053138477.1, *Streptomyces ambofaciens*).

The essential synthetic and regulatory genes of most *Streptomyces* BGCs contain transporter genes belonging to the ATP-binding cassette transporter family [19,20]. Currently, the known ABC transporters are classified into three categories according to their different structural features; these categories are Type I, Type II and Type III [21]. Although research on the ABC transporters within BGCs has been limited to date, it is generally believed that these proteins are involved in both intracellular and extracellular transport of antibiotics and production of their precursors [22]. According to Blastp and multiple sequences alignment, Atr29 contains the conserved Walker A, B motif and ABC signature motif, indicating that it belongs to the Type I sub-family of ABC transporters and might be associated with atratumycin efflux (Figure 3). Unlike the Type I ABC transporter Atr29, Atr30 belongs to the Type II sub-family of ABC transporters. Atr30 appears to be a hydrophilic protein containing nucleotide-binding domains without any hydrophobic transmembrane region and likely functions as a dimeric structure.

### 2.3. Gene Inactivation and Trans-Complementation of Regulatory and Transporter Genes

**Figure 4:**
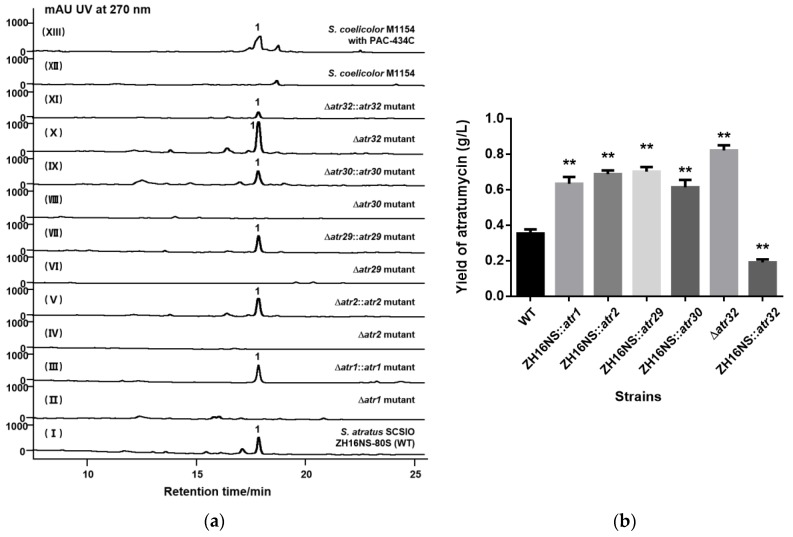
HPLC analyses of secondary metabolites. (**a**) Comparative HPLC analysis of the secondary metabolites of the wild type *S. atratus* SCSIO ZH16NS-80S (WT) with regulatory and transporter gene-complementation strains. (I) the wild type *S. atratus* SCSIO ZH16NS-80S; (II) Δ*atr1* mutant strain; (III) Δ*atr1*::*atr1*, *atr1*-complementation mutant strain; (IV) Δ*atr2* mutant strain; (V) Δ*atr2*::*atr2, atr2*-complementation mutant strain; (VI) Δ*atr29* mutant strain; (VII) Δ*atr29*::*atr29*, *atr29*-complementation mutant strain; (VIII) Δ*atr30* mutant strain; (IX) Δ*atr30*::*atr30*, *atr30*-complementation mutant strain; (X) Δ*atr32* mutant strain; (XI) Δ*atr32*::*atr32*, *atr32*-complementation mutant strain; (XII) the wild type *S. coelicolor* M1154 strain and (XIII) mutant strain *S. coelicolor* M1154 with PAC-434C. (**b**) Comparative analysis of atratumycin yield between over-expression mutants and the wild type strain (** *p* < 0.01). The values are mean ± SD from three different experiments.

Based on the above informatics analyses, five genes (*atr1*, *atr2, atr29*, *atr30* and *atr32*) were independently inactivated using λ-Red-mediated gene recombination strategies to experimentally determine the detailed function of the regulatory and transporter genes in the *atr* BGC. Double crossover mutants were verified by the phenotype and the genotype (Supplementary Materials, Figures S1–S5). High performance liquid chromatography (HPLC) analyses of the fermentation extracts clearly showed that the production of atratumycin in each of the Δ*atr1*, Δ*atr2,* Δ*atr29* and Δ*atr30* mutants was completely abolished, whereas the yield of atratumycin in the Δ*atr32* mutant was significantly improved (Figure 4a, trace X). On the basis of both bioinformatics and the inactivation experiments shown below (Figure 4a, traces VI and VII), it is apparent that Atr29 and Atr30 were involved in atratumycin secretion and/or microbial self defense mechanisms endogenous to the producer. Fascinatingly, disruption of *atr32*, which encodes a SARP family transcriptional regulator, was found to increase atratumycin titers by ca. 2.3-fold (0.823 g/L), suggesting that Atr32 is normally a suppressor of atratumycin production (Figure 4b).

To further validate the proposed functions of the *atr1*, *atr2, atr29*, *atr30* and *atr32* gene products and to disprove possible polarity effects during inactivation experiments, five integrative vectors based on pL646ATE (a pSET152-derived expression plasmid with thiostrepton and apramycin resistance genes) containing each target gene under the control of the ermE*P promoter were constructed independently [23]. The complementation mutants were verified by genotype. As a result, four mutant strains (Δ*atr1*::*atr1*, Δ*atr2*::*atr2*, Δ*atr29*::*atr29* and Δ*atr30*::*atr30*) were generated and shown to restore atratumycin production efficiencies on par with the WT producer. As noted above, atratumycin production in *S. atratus* SCSIO ZH16NS-80S::*atr32*, the complemented Δ*atr32* mutant, was found to be dramatically reduced relative to the mutant producer (Figure 4b). These data strongly suggest that, in contrast to all other regulatory/transporter genes identified in the *atr* cluster, Atr32 likely functions as a suppressor in atratumycin production.

### 2.4. Construction of the High Producing Strain by Over-Expression of Regulatory and Transporter Genes

Since Atr1 and Atr2 have been identified as pathway-specific positive regulators in the biosynthesis of atratumycin, and Atr29 and Atr30 appear to play vital roles in the secretion of atratumycin (or self-defense of the producer), we hypothesized that atratumycin titers could be improved by over-expressing these genes in *S. atratus* SCSIO ZH16NS-80S. To validate this proposal, the complementation plasmids were introduced into *S. atratus* SCSIO ZH16NS-80S by conjugation transfer and four over-expression mutants were obtained successfully. As shown in Figure 4b, the mutant strains ZH16NS-80S::*atr1* (0.635 g/L), ZH16NS-80S::*atr2* (0.690 g/L), ZH16NS-80S::*atr29* (0.704 g/L) and ZH16NS-80S::*atr30* (0.615 g/L) produced higher titers of atratumycin than the WT strain (0.355 g/L). These data further support positive regulatory roles for *atr1* and *atr2*, as well as protective roles of *atr29* and *atr30* in the biosynthesis of atratumycin. These findings lay a solid foundation for engineering strategies to optimize atratumycin production.

### 2.5. Heterologous Expression of Atratumycin

**Figure 5:**
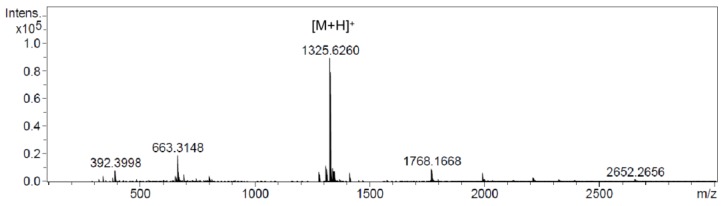
HR-ESI-MS spectra of atratumycin ([M + H]^+^ at *m*/*z* 1325.6260) of the fermentation extract of the heterologous expression mutant.

Atratumycin is a decadepsipeptide and its gene cluster is 62.7 kb in length. In order to assess the possibility that a gene cluster of this size could be heterologously expressed, a P1-derived artificial chromosome (PAC) library of the *S. atratus* SCSIO ZH16 genome was constructed using the *Escherichia coli*-*Streptomyce*s artificial chromosome vector pESAC-13-A (a derivative of pPAC-S1). This vector can be shuttled between *E. coli* and suitable *Streptomyces* hosts [24]. The PAC clone 434C containing the entire *atr* cluster was obtained and introduced into *S. coelicolor* M1154 by conjugative transfer. Three conjugants were isolated and their genotypes were confirmed by PCR analysis (Supplementary Materials, Figure S7). HPLC analysis of the crude extract produced by culturing these mutant strains under the reported atratumycin production conditions with *S. atratus* SCSIO ZH16NS-80S (Figure 4a, trace I) and *S. coelicolor* M1154 (Figure 4a, trace XII) as a control showed a new peak with a same retention time of atratumycin appeared in the recombinant strain (Figure 4a, trace XIII). The further ultraviolet absorption and high resolution electrospray ionization mass spectromerty (HR-ESI-MS) analyses revealed that the new peak in the recombinant strain has a same UV time and very close m/z (Figure 5) in *S. atratus* SCSIO ZH16NS-80S. These results indicated that the *atr* gene cluster was successfully expressed in *S. coelicolor* M1154.

## 3. Discussion

Growth periods in *Streptomyces* are characterized by cell differentiation, hyphal production and secondary metabolism; all these processes are carefully controlled by precise and complex regulatory networks [6]. Of particular interest, the biosyntheses of most secondary metabolites often display a cascade system of regulation consisting of three regulatory levels: global, pleiotropic and pathway-specific regulation. Global and pleiotropic regulatory elements are usually located outside a given BGC whereas pathway-specific regulatory factors are commonly found within specific BGCs and specifically regulate a single antibiotic biosynthetic pathway [25].

LuxR is a class of pathway-specific regulatory proteins that are involved in a variety of important physiological functions. LuxR family proteins generally act at the genetic level in *Streptomyces* and are known to regulate secondary metabolism [7]. Here, we found that Atr1 and Atr2, two LuxR-type regulators encoded within the *atr* cluster of *S. atratus* SCSIO ZH16NS-80S, positively regulate the biosynthesis of atratumycin. In parallel, SARP family proteins have long been considered to serve as positive regulators of biosynthesis in *Streptomyces* [26,27]. It is therefore highly interesting and unexpected that we have identified herein, that Atr32, a SARP representative with an NB-ARC domain, acts as a negative regulator of atratumycin assembly. The NB-ARC domain, a signaling motif found in eukaryotes and bacteria, is widely considered to be a regulatory domain that determines whether the protein is active or inactive [28,29]. Based on the sequence analyses of Atr 32 and its gene inactivation results, we speculated that the deletion of the NB-ARC domain in *atr32* prevents it from interacting with downstream signals, altering metabolic pathways and enhancing antibiotic production. This hypothesis is in line with the fact that the function of the central NB-ARC domain remains incompletely understood in *Streptomyces* sp. and requires further research.

Whole-genome sequencing initiatives have revealed that *Streptomyces* contains large numbers of transporter genes. In addition to genomics data, this conclusion is based, partly on the well-known ability of *Streptomyces* to discharge toxins, which might be their secondary metabolites or ingested toxins [30,31]. Thus far, research into ABC transporters in *Streptomyces* BGCs has been limited; most studies have only investigated whether or not ABC type transporters participate in antibiotic translocation or self-resistance processes by changing the ABC expression levels. The identification of key sites that recognize substrates, the effects of protein structure changes on transport efficiency, and regulatory patterns in ABC transport process have only just begun. Atr29 and Atr30 were both annotated as ABC transporters in the *atr* cluster and gene inactivation results strongly implicate them as key players in atratumycin secretion and/or resistance (as a means of producer self-defense). Inactivation of either Atr29 or Atr30 completely abolishes atratumycin production. Notably, the dramatic impact that Atr29 or Atr30 inactivation has on biosynthesis is not commonly seen with other ABC transporter-like systems involved in secondary metabolism [32].

*Streptomycetes* BGCs often contain both positive and negative regulatory elements. For instance, both *fkbN* (positive) and *tcs7* (negative) regulatory components exist in the FK506 biosynthetic gene cluster [33]. Not surprisingly, increasing positive and decreasing negative regulatory gene expression levels constitute effective methods for improving secondary metabolite titers [23,34,35]. Another approach commonly employed to achieve improved titers is to introduce multiple copies of positive (global or BGC specific) regulatory and/or transporter genes [36]. To some extent, the genetic tractability of the BGC-housing genomes and microbes influences the success of these types of combinatorial biosynthetic approaches. Thus, the ability to express BGCs in genetically tractable heterologous hosts determines, to a large extent, the success of titer improvement efforts based on manipulating regulatory elements. Our demonstration herein that *S. coelicolor* M1154 serves as a suitable host for over-expression of the *atr* cluster and subsequent overproduction of atratumycin therefore represents an important advancement. M1154 is a well-known and understood heterologous host for *Streptomyces*-derived BGCs. Therefore, combined with our identification and demonstrated modulation of regulatory elements of the *atr* cluster, sets the stage for further mechanistic studies of regulatory pathways employed by marine-derived secondary metabolite pathways. These advances also showcase the means by which to generate new natural products in quantities better able to support development efforts than would otherwise be possible with only WT producers; this holds true for atratumycin as well as many other secondary metabolites.

## 4. Materials and Methods 

### 4.1. General Materials and Experimental Procedures

All primers used in this studying were synthesized in Sangon Biotech Co., Ltd. (Shanghai, China) and TsingKe Biotech Co., Ltd. (Beijing, China). Molecular biology reagents were purchased from Trans Gene Co. (Beijing, China) and Takara Co. (Dalian, China) and the PCR amplifications were performed by Veriti Thermal Cycler (Applied Biosystems, Carlsbad, CA). DNA purification kit and plasmid extraction kit were purchased from Omega Biotech Co., Ltd. (Beijing, China). Antibiotics were purchased from Sangon Biotech Co., Ltd. (Shanghai, China). The media regents were purchased from Huankai Microbial Sci. & Tech, Co. Ltd. (Guangdong, China). All chemicals and solvents were purchased from standard commercial sources. All DNA manipulations and chemical experiments were conducted according to standard procedures using manufacturer protocols.

### 4.2. Bacterial Strains, Plasmids and Culture Conditions

*S. atratus* SCSIO ZH16 is a marine-derived *Streptomyces* isolated from a sediment sample collected from the South China Sea at a depth of 3536m. The construction procedure for obtaining the engineered strain *S. atratus* SCSIO ZH16NS-80S using in-frame deletion of *ilaN*-*ilaS*, atmospheric room-temperature plasma (ARTP) mutagenesis and medium optimization has been previously described [5]. *E. coli* ET12567/pUZ8002 was used as the donor cell for conjugal transfer of DNA into S. *atratus* SCSIO ZH16NS-80S and all *E. coli* strains were grown in liquid/solid LB medium (5 g/L yeast extract, 10 g/L peptone, 10 g/L NaCl) at 28 °C or 37 °C for 12–15 h. *Streptomyces* were grown on YMS medium (4 g/L yeast extract, 10 g/L malt extract, 4 g/L soluble starch, 7.5 g/L oat, 2 g/L CaCO_3_, 20 g/L agar) for the sporulation. ISP-4 medium (10 g/L soluble starch, 0.5 g/L yeast extract, 1 g/L peptone, 1 g/L K_2_HPO_4_, 1 g/L MgSO_4_•7H_2_O, 2 g/L (NH_4_)_2_SO_4_, 2 g/L CaCO_3_, 1 g/L NaCl, 20 g/L agar) supplied with 20 mM Mg^2+^ was used for the conjugation. The medium added with appropriate antibiotics at the following concentrations: 25 µg/mL chloramphenicol (Chl), 50 µg/mL apramycin (Apr), 50 µg/mL kanamycin (Kan), 50 µg/mL trimethoprim (Tmp) and 100 µg/mL ampicillin (Amp).

### 4.3. In Silico Analysis of Atratumycin Gene Cluster and Sequence Alignment of Proteins

Functional prediction and analysis of the atratumycin gene cluster was carried out using online software antiSMASH bacterial version (https://antismash.secondarymetabolites.org) and FramePlot 4.0 beta (http://nocardia.nih.go.jp/fp4/). Blastp (https://blast.ncbi.nlm.nih.gov/Blast.cgi) was used to find homologous amino acid sequences and multiple sequence alignments were carried out using the DNAMAN software package. The prediction of protein secondary structures was achieved using a number of readily available websites (https://www.genome.jp/tools/motif/, http://smart.embl-heidelberg.de/ and https://www.predictprotein.org/).

### 4.4. Construction of Gene-Inactivated Mutants

The plasmids SuperCos 1 and pIJ773 were used for S. *atratus* SCSIO ZH16 genomic library construction and the *aac(3)IV-oriT* resistance gene amplifying (Supplementary Materials, Table S1). Targeted genes in the atratumycin BGC were inactivated using λ-mediated PCR-targeting mutagenesis [5]. Three cosmids (56G, 198F and 1610C) were used to disrupt target genes. Each of the mutated genes was verified by PCR with primers designed to be 10–300 bp outside of the disruption region (Supplementary Materials, Table S2), with verification by restriction enzyme digestion. Recombination plasmids for gene replacements were further introduced into *S. atratus* SCSIO ZH16NS-80S by conjugation from *E. coli* ET12567/pUZ8002. The gene-inactivated mutant strains were selected on the basis of antibiotic selection (Apr^R^ and Kan^S^), and the genetic phenotypes of each mutant was confirmed by PCR (Supplementary Materials, Figures S1–S5).

### 4.5. Fermentation, Extraction and Quantitative Analysis

The WT *S. atratus* SCSIO ZH16NS-80S, heterologous expression strains and other mutant strains were inoculated into three 250 mL flasks per mutant with 50 mL liquid FYG medium (10 g/L fish meal, 20 g/L glycerol, 5 g/L yeast extract, 5 g/L CaCO_3_) containing 2% macroporous XAD-16 resin and incubated on a rotary shaker at 28 °C with agitation at 200 rpm. After 7 d, XAD-16 resins were separated from supernatants using rotary centrifuge at 4,000 rpm, 15min. XAD-16 resin was collected and then extracted with 100 mL EtOH. EtOH fractions were then subjected to reduced pressure to remove solvent and the remaining residues were dissolved in 2 mL methanol and centrifuged at 13,000 rpm (15 min) to achieve the clarified supernatant. These methanolic samples were then subjected to HPLC analyses. HPLC analyses were carried out using an Agilent 1260 HPLC (Agilent Technologies, Santa Clara, CA, USA) with UV detection at 270 nm at a flow rate of 1 mL/min. The mobile phase was comprised of solvents A (15% CH_3_CN in water supplemented with 0.1% acetic acid) and B (85% CH_3_CN in water supplemented with 0.1% acetic acid). Samples were eluted with a linear gradient from 0% to 80% solvent B over a duration of 20 min, followed by 80%–100% solvent B for 1.5 min, then eluted with 100% solvent B for a period of 5.5 min. The HR-ESI-MS data was obtained using an amaZon SL ion trap mass spectrometer (Bruker, Billerica, MA, USA) and quantitative analysis of atratumycin production was accomplished using GraphPad Prism 6 software. According to calibration curve, the quantification of atratumycin yields was determined (Supplementary Materials, Figure S6).

### 4.6. Complementation and Over-Expression in Mutants or the WT Producer Strain

The coding regions of *atr1*, *atr2*, *atr29*, *atr30* and *atr32* were amplified by PCR using the genomic DNA of *S. atratus* SCSIO ZH16NS and all primers are listed in Table S2. Each of the PCR products was digested with *Nde*I/*Spe*I then cloned into pL646ATE with the same restriction sites, a pSET152-derived expression plasmid with same digested sites. The recombinant plasmids pL646ATE-*atr1*, pL646ATE-*atr2*, pL646ATE-*atr29*, pL646ATE-*atr30* and pL646ATE-*atr32* were transformed into *E. coli* ET12567/pUZ8002 for complementation and over-expression experiments in the *S. atratus* ZH16NS-80S WT strain and mutant strains. Exconjugants were selected based on phenotypes showing thiostrepton resistance and then confirmed by PCR. The quantitative HPLC standard curve for atratumycin was drawn using origin software (Supplementary Materials, Figure S6).

### 4.7. Construction of Genomic PAC Library and Heterologous Expression of the Atr Gene Cluster

The PAC library of *S. atratus* SCSIO ZH16NS was constructed using pESAC13-A from Bio S&T (Montreal, Canada). The integrative vector pESAC13-A is an *E. coli–Streptomyces* artificial chromosome with an *oriT* site that allows transfer into *Streptomyces* by conjugation and confers apramycin resistance in both *E. coli* and *Streptomyces*. Three sets of primers, including ID-*atr19*-F/R, ID-*orf(-2)*-F/R, and ID-*orf(+2)*-F/R, were designed to screen the library for the desired clone that contained all *atr* biosynthetic genes (Supplementary Materials, Table S2). PAC-434C containing the entire gene cluster was screened out (Supplementary Materials, Figure S7) and transferred into *S. atratus* SCSIO ZH16NS-80S or mutant strains by intergeneric conjugal transfer [37].

## Supplementary Materials

The following are available online at https://www.mdpi.com/1660-3397/17/10/560/s1, Table S1: Summary of strains and plasmids used in this study, Table S2: Summary of primers used in this study, Figures S1–S5: Disrupted genes in the atr cluster in the WT SCSIO ZH16NS-80S achieved via PCR-targeting, Figure S6: The quantitative HPLC standard curve for atratumycin, Figure S7: PCR verification of PAC434 containing the entire *atr* gene cluster.
