# Supplementary material for: Characterization of the Noncanonical Regulatory and Transporter Genes in Atratumycin Biosynthesis and Production in a Heterologous Host
Source: Mar Drugs. 2019 Sep 29;17(10):560. doi: 10.3390/md17100560 (PMC6835768; doi:10.3390/md17100560)
Supplement: Supplementary file 1 [file marinedrugs-17-00560-s001.pdf]

Supplementary Materials

Characterization of the Noncanonical Regulatory and Transporter Genes in Atratumycin Biosynthesis and Production in a Heterologous Host

Zhijie Yang [1,2], Xin Wei [1,2], Jianqiao He [1,2], Changli Sun [1], Jianhua Ju [1,2,*] and Junying Ma [1,*]

[1] CAS Key Laboratory of Tropical Marine Bio-resources and Ecology, Guangdong Key Laboratory of Marine Materia Medica, RNAM Center for Marine Microbiology, South China Sea Institute of Oceanology, Chinese Academy of Sciences, Guangzhou, 510301, China

[2] College of Earth and Planetary Sciences, University of Chinese Academy of Sciences, Beijing, 100049, China.

[*] Correspondence: jju@scsio.ac.cn; Tel.: (+86)20-89023028 (J.J.); majunying@scsio.ac.cn; Tel.: (+86)20-34066449 (J.M.)

Item Name	Contents/Description	Page
**Table S1**	Summary of strains and plasmids used in this study.	S1-S2
**Table S2**	Summary of primers used in this study.	S2-S3
**Figure S1**	Disruption of *atr1* in WT *S. atratus* SCSIO ZH16NS-80S.	S3
**Figure S2**	Disruption of *atr2* in WT *S. atratus* SCSIO ZH16NS-80S.	S4
**Figure S3**	Disruption of *atr29* in WT *S. atratus* SCSIO ZH16NS-80S.	S4
**Figure S4**	Disruption of *atr30* in WT *S. atratus* SCSIO ZH16NS-80S.	S5
**Figure S5**	Disruption of *atr32* in WT *S. atratus* SCSIO ZH16NS-80S.	S5
**Figure S6**	The quantitative HPLC standard curve for atratumycin.	S6
**Figure S7**	PCR verification of PAC434 containing entire *atr* gene cluster.	S6

Table S1. Summary of strains and plasmids used in this study.

Strains/ plasmids	Relevant phenotype	Source/ [Ref]
S. atratus SCSIO ZH16NS-80S	Wild-type (WT) producer of atartumycin	[1]
Δ*atr1*	*S. atratus* SCSIO ZH16NS with a 624 bp of *atr1* substituted by *aac(3)IV*+OriT	This work
Δ*atr2*	*S. atratus* SCSIO ZH16NS with a 315 bp of *atr2* substituted by *aac(3)IV*+OriT	This work
Δ*atr29*	*S. atratus* SCSIO ZH16NS with a 729 bp of *atr29* substituted by *aac(3)IV*+OriT	This work
Δ*atr30*	*S. atratus* SCSIO ZH16NS with a 447 bp of *atr30* substituted by *aac(3)IV*+OriT	This work
Δ*atr32*	*S. atratus* SCSIO ZH16NS with a 612 bp of *atr32* substituted by *aac(3)IV*+OriT	This work
E. coli		
Bw25113	K-12 derivative: *araBAD, rhaBAD*	[2]
ET12567	*dam, dcm, hsdM, hsdS, hsdR, catR , tetR*	[3]
Plasmids		
pIJ773	P1-FRT-oriT-aac(3)IV-FRT-P2	[4]
pIJ790	λ-RED (*gam bet exo*) CmlR *araCrep101ts*	[4]
pUZ8002	*tra, neo,* RP4	[5]
pL646ATE	*Tsr, acc(3)IV, ermE*P*	[6]
cosmid-56G	A cosmid which contains partial atratumycin biosynthesis cluster	This work
cosmid-198F	A cosmid which contains partial atratumycin biosynthesis cluster	This work
cosmid-1610C	A cosmid which contains partial atratumycin biosynthesis cluster	This work
PAC-434C	A cosmid which contains complete actinomycin biosynthesis cluster	This work
*p*Δ*atr1*	A 624 bp fragment in *atr1* in cosmid 56G was substituted by the aac(IV)+OriT cassette using the PCR-targeting strategy	This work
*p*Δ*atr2*	A 315 bp fragment in *atr2* in cosmid 56G was substituted by the aac(IV)+OriT cassette using the PCR-targeting strategy	This work
*p*Δ*atr29*	A 729 bp fragment in *atr29* in cosmid 1610C was substituted by the aac(IV)+OriT cassette using the PCR-targeting strategy	This work
*p*Δ*atr30*	A 447 bp fragment in *atr30* in cosmid 1610C was substituted by the aac(IV)+OriT cassette using the PCR-targeting strategy	This work
*p*Δ*atr32*	A 612 bp fragment in *atr32* in cosmid 1610C was substituted by the aac(IV)+OriT cassette using the PCR-targeting strategy	This work
ZH16NS-80S::atr1	An integrated vector pL646ATE with complete *atr1* for over-expression	This work

ZH16NS-80S::atr2	An integrated vector pL646ATE with complete *atr2* for over-expression	This work
ZH16NS-80S::atr29	An integrated vector pL646ATE with complete *atr29* for over-expression	This work
ZH16NS-80S::atr30	An integrated vector pL646ATE with complete *atr30* for over-expression	This work
ZH16NS-80S::atr32	An integrated vector pL646ATE with complete *atr32* for over-expression	This work

Table S2. Summary of primers used in this study.

Primer Name	Sequence (5'→3')	purpose
DEL-*atr1*-F	gaaagcgcgttgcttcaggcccgtgcgctgatcgaatcgattccgg ggatccgtcgacc	For disrupting *atr1*
DEL-*atr1*-R	ctggaaacgggagttggccccgagctcgcgcatgatctctgtaggc tggagctgcttc	
DEL-*atr2*-F	gaacttcggctcctgtgcacccaggcgatgctcgacgacattccggg gatccgtcgacc	For disrupting *atr2*
DEL-*atr2*-R	gacgtaccggcgataggtgcgcaccgagacggagagctctgtagg ctggagctgcttc	
DEL-*atr29*-F	gagggaacggtcctcggactgctcggccccaacggtgcgattccg gggatccgtcgacc	For disrupting *atr29*
DEL-*atr29*-R	gcgccgaacggcgaactccgctatgtcgattccgagttctgtaggct ggagctgcttc	
DEL-*atr30*-F	atgatggtcaacatctcccagacggccctgggcgtcgtcattccggg gatccgtcgacc	For disrupting *atr30*
DEL-*atr30*-R	ccccgtaccgatgcccacggtgggattgccgaagagttgtgtaggc tggagctgcttc	
DEL-*atr32*-F	gccaccgcgacggcccagatctacacctatgtctcccagattccggg gatccgtcgacc	For disrupting *atr32*
DEL-*atr32*-R	catcgcgggcaggacgccgctccaggcgccgcggtcccgtgtagg ctggagctgcttc	
COM-*atr1*-F	aaaacatatgatgaagacttccacgtctaaaac	For cloning complete
COM-*atr1*-R	aaaaactagttcatgacgtcgtgattctctg	*atr1*
COM-*atr2*-F	aaaacatatgatgaccgattcctattctgtc	For cloning complete
COM-*atr2*-R	aaaaactagtctacaggaagctgccagtgc	*atr2*
COM-*atr29*-F	aaaacatatgatgacgaacgccatcgaggc	For cloning complete
COM-*atr29*-R	aaaaactagttcacgccgattccctctccc	*atr29*
COM-*atr30*-F	aaaacatatggtgaccgagacactcagtgac	For cloning complete
COM-*atr30*-R	aaaaactagttcagcgagcggtcgccg	*Atr30*
COM-*atr32*-F	aaaacatatggtgaacggcttcgaccc	For cloning complete
COM-*atr32*-R	aaaaactagtctacatcgactccctga	*Atr32*
ID-*atr1*-F	gccaataccgccgaaggcatc	For verifying mutant
ID-*atr1*-R	cagacccatctccacggctc	Δ*atr1*
ID-*atr2*-F	cggaagcagatcgaacttcgg	For verifying mutant
ID-*atr2*-R	caggagcgccatgatctccgc	Δ*atr2*
ID-*atr29*-F	gccatcgaggccgaaggcctc	For verifying mutant
ID-*atr29*-R	gacgtcgtcgagcgaagggcg	Δ*atr29*

ID-*atr30*-F	caggagtacatcatggcgggc	For verifying mutant Δ*atr30*
ID-*atr30*-R	cagcgacatcacgatggggtg	
ID-*atr32*-F	gaagcagcgcaccgtcctcg	For verifying mutant Δ*atr32*
ID-*atr32*-R	gacttgccggtacctgcagc	
ID-*atr19*-F	gcgggtggacctggcgaag	For screening PAC clones containing *atr* BGC
ID-*atr19*-R	gcagccgtcgggagcgacg	
ID-*orf(-2)*-F	cccatacgccgctacgaacgc	For screening PAC clones containing *atr* BGC
ID-*orf(-2)*-R	ccgttgcagtggcacgagttc	
ID-*orf(+2)*-F	gaaatggtgctggaggtcacg	For screening PAC clones containing *atr* BGC
ID-*orf(+2)*-R	ctagatccgccgctccgcacc	
ID-com-F	gatctgaccgacgcggtccac	For verifying complete gene fragments
ID-com-R	gcgcggtcgatccccgcatg	

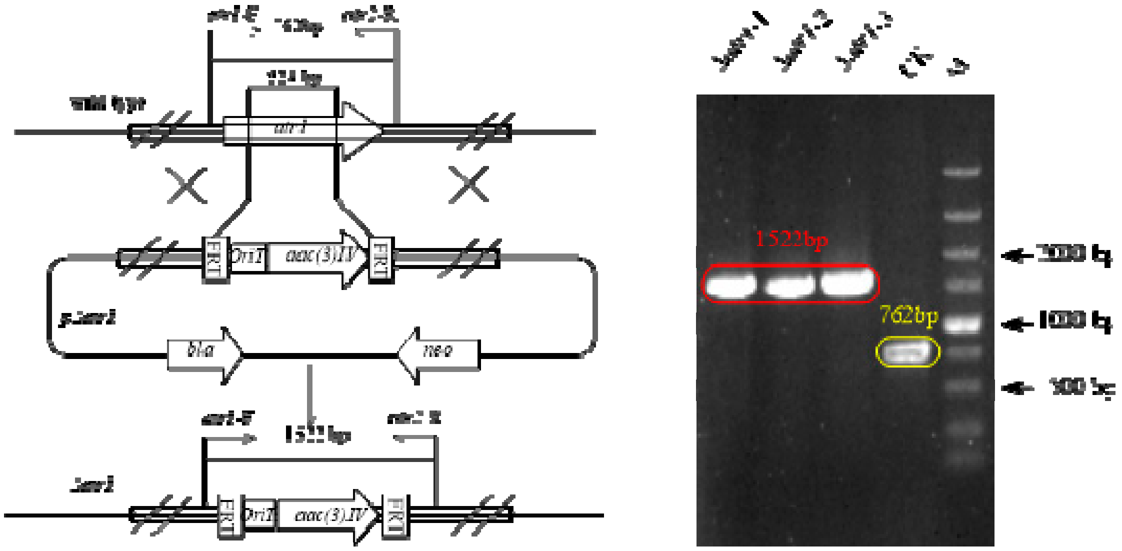

(**a**) (**b**)

Figure S1. Disruption of *atr1* in *S. atratus* SCSIO ZH16NS-80S via PCR-targeting. **a**) Schematic representation for disruption of *atr1*. **b**) PCR analysis of the control strain (*S. atratus* SCSIO ZH16NS-80S) and the *atr1* mutants carried out using primers listed in **Table S2**. M: DNA molecular ladder; CK: using the genomic DNA of *S. atratus* SCSIO ZH16NS-80S as template, Δ*atr1* (1-3): using the genomic DNA of three different colonies of Δ*atr1* mutant as template.

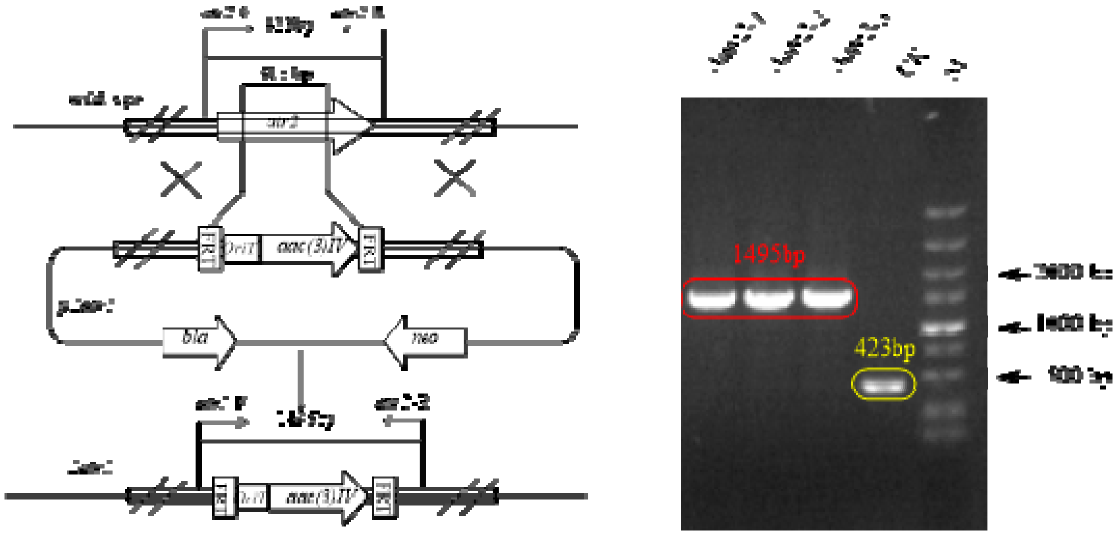

(a) (b)

Figure S2. Disruption of *atr2* in *S. atratus* SCSIO ZH16NS-80S via PCR-targeting. **a**) Schematic representation for disruption of *atr2*. **b**) PCR analysis of the control strain (*S. atratus* SCSIO ZH16NS-80S) and the *atr2* mutants carried out using primers listed in **Table S2**. M: DNA molecular ladder; CK: using the genomic DNA of *S. atratus* SCSIO ZH16NS-80S as template, Δ*atr2* (1-3): using the genomic DNA of three different colonies of Δ*atr2* mutant as template.

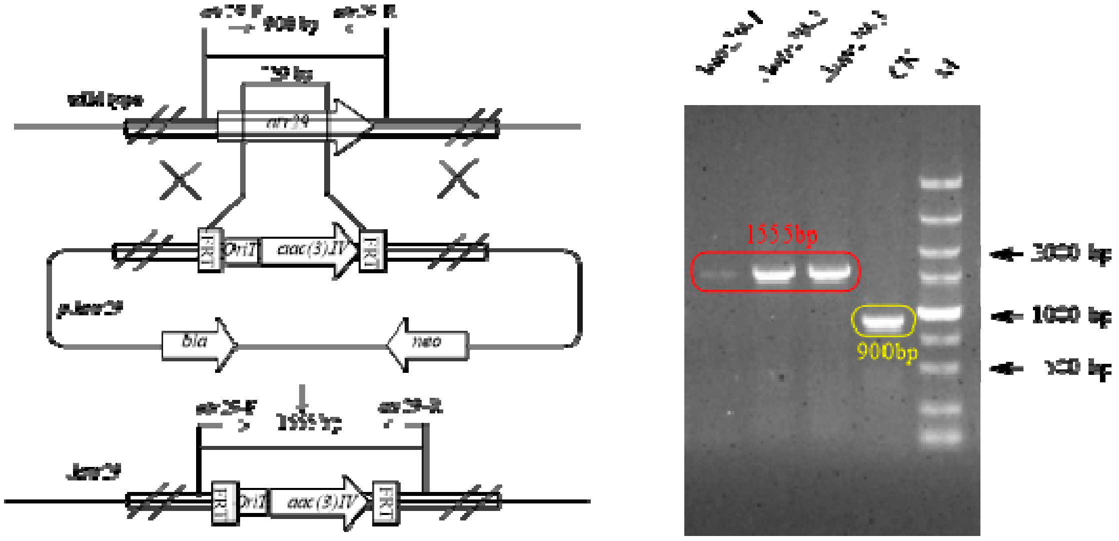

(a) (b)

Figure S3. Disruption of *atr29* in *S. atratus* SCSIO ZH16NS-80S via PCR-targeting. **a**) Schematic representation for disruption of *atr29*. **b**) PCR analysis of the control strain (*S. atratus* SCSIO ZH16NS-80S) and the *atr29* mutants carried out using primers listed in **Table S2**. M: DNA molecular ladder; CK: using the genomic DNA of *S. atratus* SCSIO ZH16NS-80S as template, Δ*atr29* (1-3): using the genomic DNA of three different colonies of Δ*atr29* mutant as template.

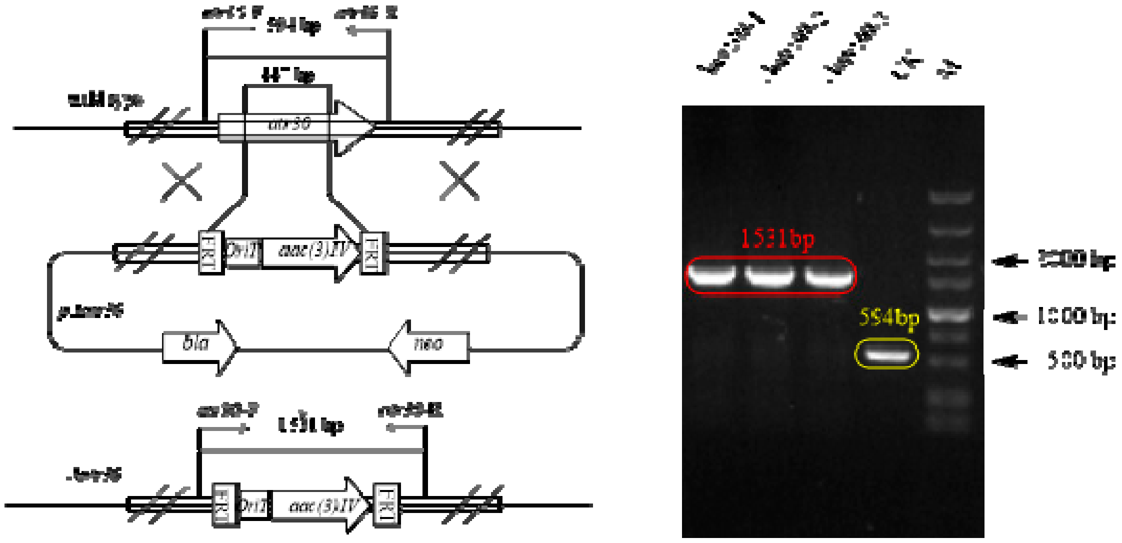

(a) (b)

Figure S4. Disruption of *atr30* in *S. atratus* SCSIO ZH16NS-80S via PCR-targeting. **a**) Schematic representation for disruption of *atr30*. **b**) PCR analysis of the control strain (*S. atratus* SCSIO ZH16NS-80S) and the *atr30* mutants carried out with the primers listed in **Table S2**. M: DNA molecular ladder; CK: using the genomic DNA of *S. atratus* SCSIO ZH16NS-80S as template, Δ*atr30* (1-3): using the genomic DNA of three different colonies of Δ*atr30* mutant as template.

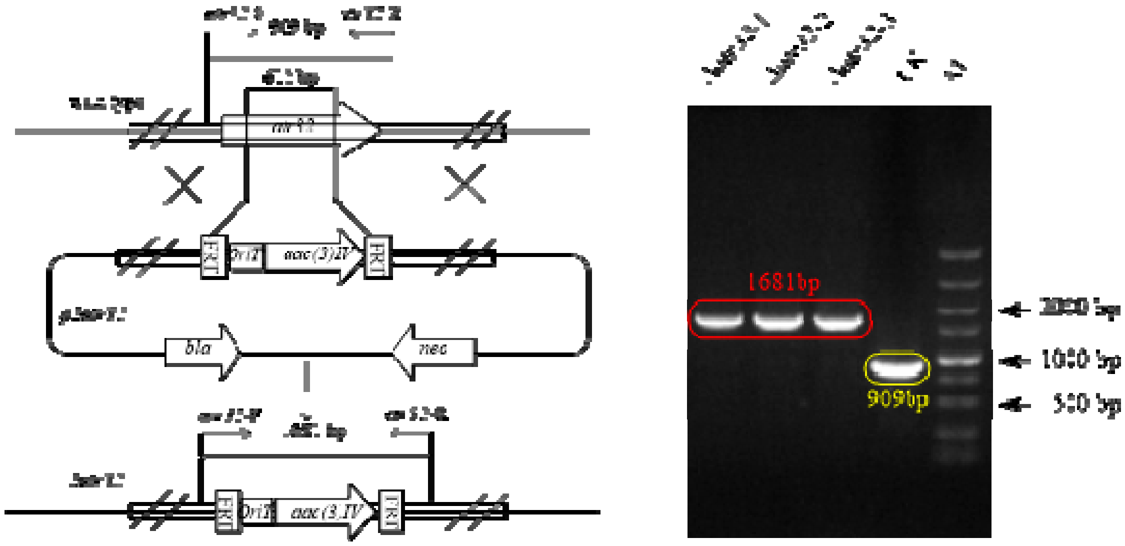

(a) (b)

Figure S5. Disruption of *atr32* in *S. atratus* SCSIO ZH16NS-80S via PCR-targeting. **a**) Schematic representation for disruption of *atr32*. **b**) PCR analysis of the control strain (*S. atratus* SCSIO ZH16NS-80S) and the *atr32* mutants carried out using primers listed in **Table S2**. M: DNA molecular ladder; CK: using the genomic DNA of *S. atratus* SCSIO ZH16NS-80S as template, Δ*atr32* (1-3): using the genomic DNA of three different colonies of Δ*atr32* mutant as template.

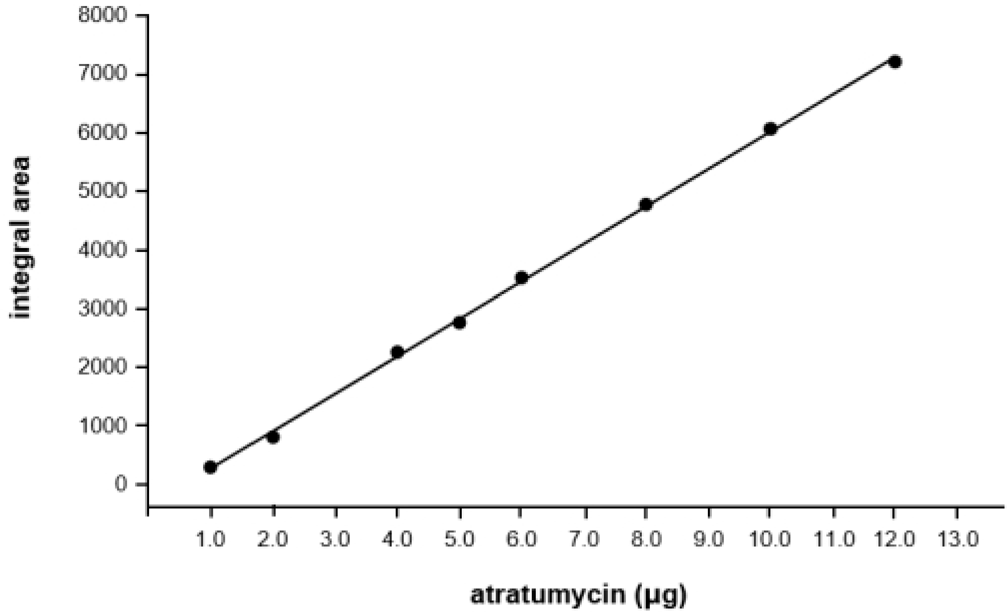

Figure S6. The quantitative HPLC standard curve for atratumycin. The curve was generated via analysis of a concentration gradient of 1.0 μg, 2.0 μg, 4.0 μg, 5.0 μg, 6.0 μg, 8.0 μg, 10.0 μg, 12.0 μg of atratumycin. UV absorption was maintained below 1 A unit to ensure appropriate confidence of the generated standard curve and avoid deviation from linearity.

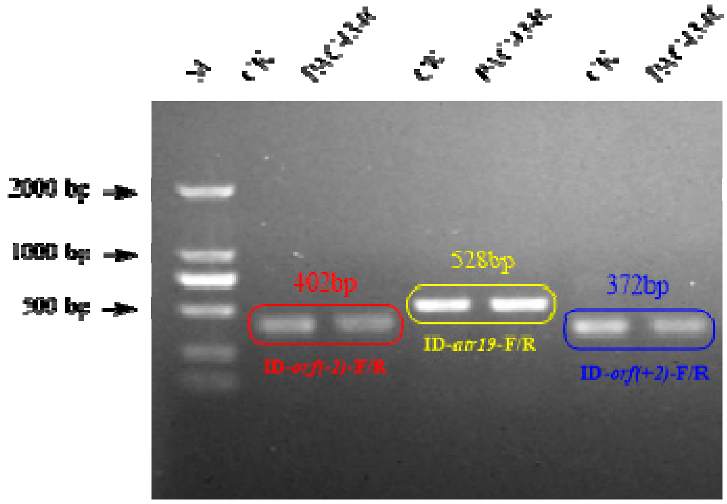

Figure S7. PCR verification of PAC434 containing entire *atr* gene cluster. M: DNA molecular ladder; CK: using the genomic DNA of *S. atratus* SCSIO ZH16NS-80S as template.

References

1. Sun, C.; Yang, Z.; Zhang, C.; Liu, Z.; He, J.; Liu, Q.; Zhang, T.; Ju, J.; Ma, J., Genome Mining of *Streptomyces atratus* SCSIO ZH16: Discovery of Atratumycin and Identification of Its Biosynthetic Gene Cluster. *Organic Letters* **2019**, 21, (5), 1453-1457.

2. Macneil, D. J.; Gewain, K. M.; Ruby, C. L.; Dezeny, G.; Gibbons, P. H.; Macneil, T., Analysis of *Streptomyces avermitilis* genes required for avermectin biosynthesis utilizing a novel integration vector. *Gene* **1992**, 111, (1), 61-68.
3. Datsenko, K. A.; Wanner, B. L., One-step inactivation of chromosomal genes in Escherichia coli K-12 using PCR products. *Proceedings of the National Academy of Sciences of the United States of America* **2000**, 97, (12), 6640-6645.
4. Gust, B.; Chandra, G.; Jakimowicz, D.; Tian, Y. Q.; Bruton, C. J.; Chater, K. F., lambda red-mediated genetic manipulation of antibiotic-producing *Streptomyces*. *Advances in Applied Microbiology*, Vol 54 **2004**, 54, 107-128.
5. Paget, M. S. B.; Chamberlin, L.; Atrih, A.; Foster, S. J.; Buttner, M. J., Evidence that the extracytoplasmic function sigma factor sigma(E) is required for normal cell wall structure in *Streptomyces coelicolor* A3(2). *Journal of Bacteriology* **1999**, 181, (1), 204-211.
6. Tu, J.; Li, S.; Chen, J.; Song, Y.; Fu, S.; Ju, J.; Li, Q., Characterization and heterologous expression of the neoabyssomicin/abyssomicin biosynthetic gene cluster from *Streptomyces koyangensis* SCSIO 5802. *Microbial Cell Factories* **2018**, 17, (28), 1-14.
